# Ion-sensing properties of 1D vanadium pentoxide nanostructures

**DOI:** 10.1186/1556-276X-7-310

**Published:** 2012-06-18

**Authors:** Nirton CS Vieira, Waldir Avansi, Alessandra Figueiredo, Caue Ribeiro, Valmor R Mastelaro, Francisco EG Guimarães

**Affiliations:** 1 Departamento de Física e Ciências dos Materiais, Instituto de Física de São Carlos, Universidade de São Paulo, Avenida Trabalhador São-carlense 400, São Carlos, São Paulo, CP 369/13560-970, Brazil; 2 Departamento de Físico-Química, Instituto de Química de Araraquara, Universidade Estadual Paulista Júlio de Mesquita Filho, Rua Prof. Francisco Degni 55, Araraquara, São Paulo, CP 355/14801-907, Brazil; 3 Embrapa, Empresa Brasileira de Pesquisas Agropecuárias, Rua XV de Novembro 1452, São Carlos, São Paulo, 13560-970, Brazil

**Keywords:** Vanadium pentoxide, Nanostructures, pH sensors, SEGFET, Hydrothermal synthesis

## Abstract

The application of one-dimensional (1D) V_2_O_5_·*n*H_2_O nanostructures as pH sensing material was evaluated. 1D V_2_O_5_·*n*H_2_O nanostructures were obtained by a hydrothermal method with systematic control of morphology forming different nanostructures: nanoribbons, nanowires and nanorods. Deposited onto Au-covered substrates, 1D V_2_O_5_·*n*H_2_O nanostructures were employed as gate material in pH sensors based on separative extended gate FET as an alternative to provide FET isolation from the chemical environment. 1D V_2_O_5_·*n*H_2_O nanostructures showed pH sensitivity around the expected theoretical value. Due to high pH sensing properties, flexibility and low cost, further applications of 1D V_2_O_5_·*n*H_2_O nanostructures comprise enzyme FET-based biosensors using immobilized enzymes.

## Background

Proton donor-acceptor property (amphoterism) is characteristic of several metal oxides or nitrides. These properties have enabled the development of numerous devices to measure ion activities in chemical environments, including ion-sensitive field-effect transistors (ISFET) [1], capacitive electrolyte-insulator-semiconductors [2], light-addressable potentiometric sensors [3], and separative extended gate field-effect transistors (SEGFET) [4]. All these devices are based on field effect and the surface potential of gate insulator material that changes according to the ion concentration in the solution, controlling the output signal. ISFET is the most common type of field-effect device used in pH sensors and biosensors because it can be miniaturized and manufactured on a large scale. However, in ISFET sensors, the FET is in direct contact with the solution, which can hinder the measurement and immobilization of biomolecules due to their small dimensions. As an alternative, a SEGFET [4] or, in a simple way, a sensitive layer connected to the input pin of a high-impedance buffer, such as an operational amplifier [5,6], can be utilized. In both cases, the transduction principle (field effect) is the same. Besides the reuse of the FET in new measurements, the robustness and flexibility of the extended sensitive layer facilitate the processing of new materials to be implemented as ion sensors.

Since the technology of field-effect devices is mature, research has focused on the synthesis of new materials to be applied as ion sensitive membranes. Several metal oxides or nitrides that have been used as pH sensitive membranes have presented the expected response [7-10]. In fact, nanoscale metal oxides can improve the fundamental properties of materials and the performance of devices due to new physical and chemical properties. Recently, one-dimensional (1D) nanostructured materials such as nanowires, nanoribbons and nanotubes have attracted much interest due to their improved properties when compared to similar isotropic nanostructures [11-13].

Vanadium pentoxide (V_2_O_5_), which possesses particularly interesting physical and chemical properties, has been employed in technological applications as catalytic material [14], in electrochromic devices [15], as battery cathode material [16], and in sensors [17-19]. Several strategies have been developed to obtain 1D V_2_O_5_ nanostructures. For example, Avansi et al. recently reported an environmentally correct, one-step hydrothermal route for the synthesis of V_2_O_5_·*n*H_2_O nanostructures with controlled morphology and crystalline structure [20].

Combining SEGFET devices and V_2_O_5_·*n*H_2_O nanostructures, field-effect sensors can be constructed in a simple and low-cost way. In this context of technological applications, we report on the use of 1D V_2_O_5_·*n*H_2_O nanostructures obtained by a hydrothermal method as pH sensitive membranes in a SEGFET device, which was constructed based on van der Spiegel’s concept [5].

## Methods

The V_2_O_5_·*n*H_2_O nanostructures were synthesized by a hydrothermal method which is described in detail elsewhere [20]. Briefly, this procedure involves dissolving V_2_O_5_ micrometric powder (Alfa Aesar, Ward Hill, MA, USA; 99.995% purity) in deionized water, adding hydrogen peroxide (H_2_O_2_), and treating the mixture hydrothermally. Different V_2_O_5_·*n*H_2_O 1D nanostructures were obtained by applying the hydrothermal treatment at different temperatures in the same time of synthesis (24 h) [20].

The crystalline phase of the as-obtained samples was investigated by X-ray diffraction (XRD) using a Shimadzu XRD 6000 diffractometer (Shimadzu Corporation, Nakagyo-ku, Kyoto, Japan) with Cu *kα* (*λ* = 1.5406) radiation. The size and morphology of the as-obtained samples were determined using a Zeiss VP Supra 35 field emission scanning transmission electron microscope (FE-STEM; Carl Zeiss AG, Oberkochen, Germany).

**Figure 1:**
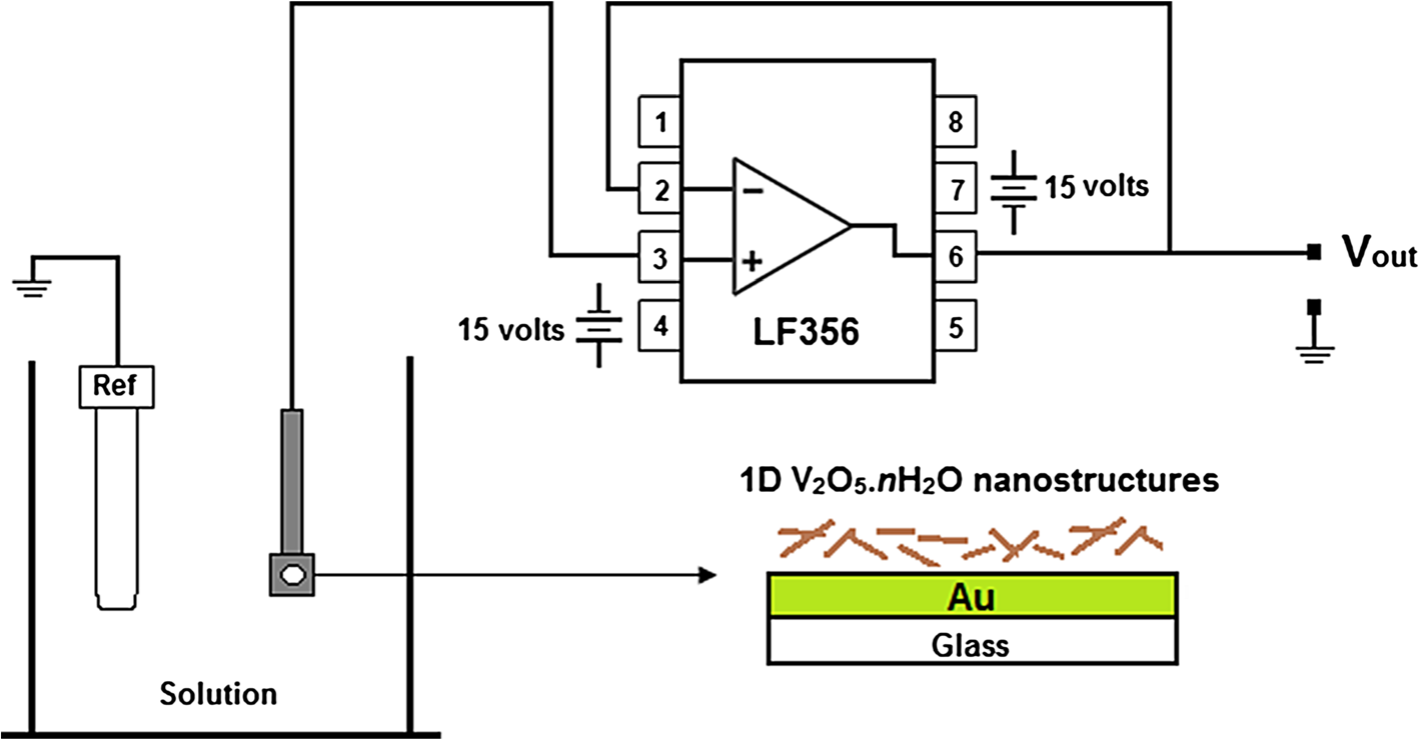
**Schematic diagram of the SEGFET configuration.** The electronic diagram of LF356 operational amplifier is shown.

The as-obtained samples were deposited onto Au-coated substrates by spin coating and connected to the input pin of a LF356 JFET operational amplifier, used here as a unity gain buffer. A silver/silver chloride (Ag/AgCl) reference electrode was used to keep the voltage constant. Figure 1 shows a schematic diagram of the SEGFET.

## Results and discussion

**Figure 2:**
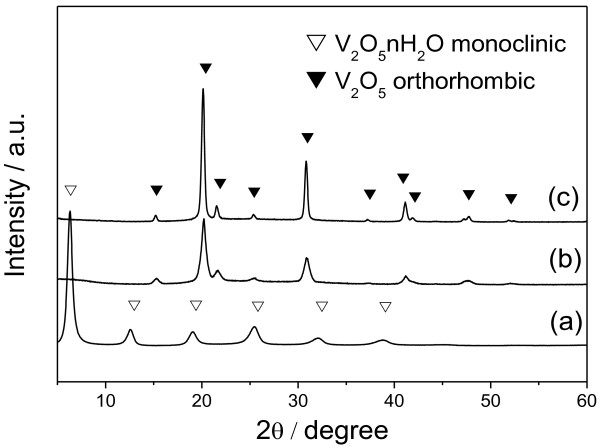
**XRD diffractograms of the samples synthesized by the hydrothermal route.** (**a**) Nanoribbon at 160°C, (**b**) nanowire at 180°C and (**c**) nanorod at 200°C.

The diffractograms in Figure 2 confirm the expected crystalline phase in all the samples under study, i.e., monoclinic phase in the samples synthesized at 160°C and orthorhombic phase in those synthesized at 180°C and 200°C [20].

**Figure 3:**
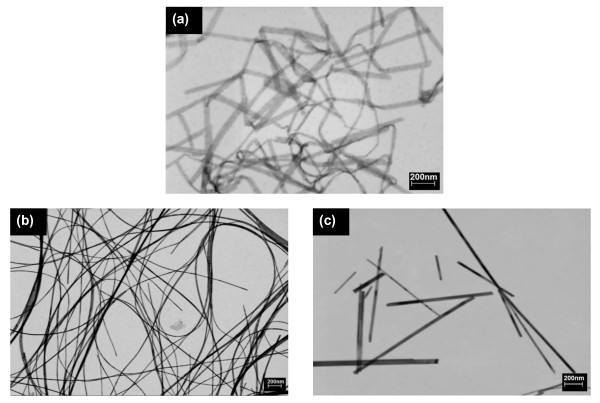
**FE-STEM images of a 1D V**_**2**_**O**_**5**_**.*n*H**_**2**_**O nanostructures synthesized.** (**a**) 160°C, (**b**) 180°C and (**c**) 200°C.

The bright field scanning transmission electron microscopy (STEM) images shown in Figure 3 confirm the morphology of the resulting nanostructures. As expected, different nanostructures were obtained. The samples synthesized at 160°C show a nanoribbon-like morphology (Figure 3a), while samples synthesized at 180°C and 200°C present, respectively, nanowire-like (Figure 3b) and nanorod-like (Figure 3c) morphologies [20].

SEGFET devices have been used as an alternative to conventional ISFET to isolate FET from analytical chemical environments and have presented the same operational characteristics [4,6,9,18]. The robustness and flexibility of the gate in SEGFET devices allow for the combination and testing of new materials that can sense pH easily. In addition, the commercial high-input impedance device (FET part) in SEGFET sensors can be reused, since only the extended gate membrane has to be built [4,6,9,18].

**Figure 4:**
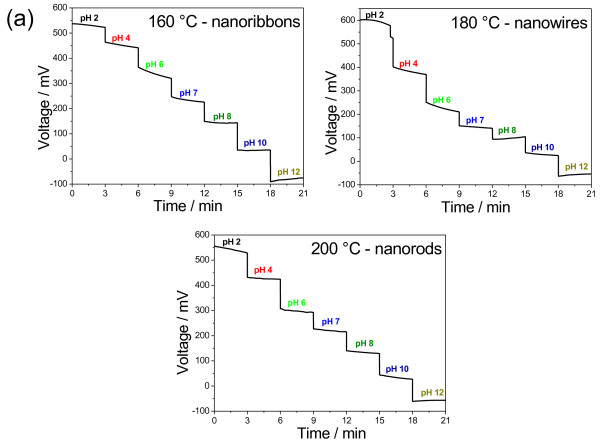
**Dynamic response of all 1D V**_**2**_**O**_**5**_**·*n*H**_**2**_**O nanostructures to pH variations.** (**a**) Typical dynamic response of 1D V_2_O_5_·*n*H_2_O nanostructured sensing membranes to variations in pH and (**b**) pH sensitivity calculated at 3 min. Inset: pH sensitivity of 1D V_2_O_5_·*n*H_2_O nanostructures as a function of hydrothermal synthesis temperature.

The 1D V_2_O_5_·*n*H_2_O nanostructures deposited on Au-coated substrates were immersed in buffer solutions with different pH (pH from 2 to 12), and the output voltage of the operational amplifier was recorded over time. Figure 4a shows the dynamic response of all 1D V_2_O_5_·*n*H_2_O nanostructures to pH variations. Despite the structural changes due to the conditions of hydrothermal synthesis, the V_2_O_5_·*n*H_2_O synthesized at 160°C (in nanoribbon form with monoclinic phase) and at 180°C (in nanowire form with orthorhombic phase) yielded similar results. The pH sensitivity of the 1D V_2_O_5_·*n*H_2_O nanostructures was determined based on the output voltage at 3 min. Within the limits of experimental error, the sensitivity did not change in any of the V_2_O_5_·*n*H_2_O morphologies, indicating that the pH sensitivity is independent of the phase or nanostructure, as indicated in the inset in Figure 4b.

The mechanism of pH sensitivity is due to the amphoteric properties of the majority of metal oxides and can be explained by the well-known site-binding model [21,22]. According to this model, the surface of V_2_O_5_·*n*H_2_O nanostructures contains three sites, i.e., negatively charged groups, neutral groups and positively charged groups. The total surface charge can be altered by the formation of metal complexes on the surface of V_2_O_5_·*n*H_2_O nanostructures according to the following equation [21,22]:

(1) $$\psi =\frac{2\text{,3k}T}{q}\frac{\beta }{\beta +1}{(\text{pH}}_{\text{pzc}}-\text{ pH})$$

where pH_pzc_ is the pH value at the point of zero charge, *q* is the elementary charge, k is the Boltzmann constant, *T* is the absolute temperature, and *β* is a factor that reflects the chemical sensitivity of the gate material. Modifications in the pH of the electrolyte cause changes in the concentration of protons, allowing for control of the output signal of SEGFET devices. The site-binding model is consistent with the experimental results, indicating that the value of *β* is the same for any V_2_O_5_·*n*H_2_O morphologies.

The pH sensitivity of 1D V_2_O_5_·*n*H_2_O nanostructures is consistent with the theoretical Nernstian value expected for pH-sensitive materials (59.2 mV.pH^^−1^) and in excellent agreement with values reported for other metal oxide pH-sensing membranes [6-10]. In addition, due to this property, 1D V_2_O_5_·*n*H_2_O nanostructures can be applied as field-effect based biosensors, since the biomolecule-catalyzed reaction changes the ion concentration in solution, as suggested in the literature [23].

## Conclusions

In summary, we have reported the results of an investigation of vanadium pentoxide nanostructures as sensitive material in SEGFET pH sensors. The use of the hydrothermal route combined with FET-based sensors yielded nanometric pH-sensitive materials. 1D V_2_O_5_·*n*H_2_O nanostructures showed pH sensitivity close to the theoretical value. Despite the influence of the synthesis temperature on the morphological and structural properties of the material, its pH sensitivity remained unaffected, as expected. Our strategy shows potential advantages for the construction of low-cost pH sensing membranes with promising applications in field effect-based biosensors.

## Competing interests

The authors declare that they have no competing interests.

## Authors’ contributions

NCSV conceived the study, contributed with its design and coordination, and drafted the manuscript. WA, CR and VRM synthesized all vanadium pentoxide nanostructures, and they were responsible for its characterization. AF made the films and helped the experiments related to the pH sensor. FEGG gave advice and guided the experiments. All authors read and approved the final manuscript.
